# *Wwox* Deletion in Mouse B Cells Leads to Genomic Instability, Neoplastic Transformation, and Monoclonal Gammopathies

**DOI:** 10.3389/fonc.2019.00517

**Published:** 2019-06-19

**Authors:** Kevin M. McBride, Hyunsuk Kil, Yunxiang Mu, Joshua B. Plummer, Jaeho Lee, Maciej J. Zelazowski, Manu Sebastian, Martin C. Abba, C. Marcelo Aldaz

**Affiliations:** ^1^Department of Epigenetics and Molecular Carcinogenesis, Science Park, The University of Texas MD Anderson Cancer Center, Smithville, TX, United States; ^2^School of Medicine, Center for Immunological Basic and Applied Research (CINIBA), National University of La Plata (UNLP), La Plata, Argentina

**Keywords:** Wwox, B cells, monoclonal gammopathies, plasmacytomas, multiple myeloma, genomic instability

## Abstract

*WWOX* (WW domain containing oxidoreductase) expression loss is common in various cancers and characteristic of poor prognosis. Deletions, translocations, and loss of expression affecting the *WWOX* gene are a common feature of various B cell neoplasms such as certain B cell lymphomas and multiple myeloma. However, the role of this common abnormality in B cell tumor initiation and/or progression has not been defined. In this study, we conditionally deleted *Wwox* early in B cell development by means of breeding *Cd19-Cre* transgenic mice crossed to *Wwox* floxed mice (*Cd19 Wwox KO*). We observed a significant reduced survival in *Cd19 Wwox KO* mice and the development of B cell neoplasms including B cell lymphomas, plasma cell neoplasias characterized by increased numbers of CD138+ populations as well as monoclonal gammopathies detected by serum protein electrophoresis. To investigate whether *Wwox* loss could play a role in genomic instability, we analyzed DNA repair functions during immunoglobulin class switch joining between DNA segments in antibody genes. While class switch recombination (CSR) was only slightly impaired, Wwox deficiency resulted in a dramatic shift of double strand break (DSB) repair from normal classical-NHEJ toward the microhomology-mediated alternative-NHEJ pathway, a pathway associated with chromosome translocations and genome instability. Consistent with this, Wwox deficiency resulted in a marked increase of spontaneous translocations during CSR. This work defines for the first time a role for Wwox for maintaining B cell genome stability during a process that can promote neoplastic transformation and monoclonal gammopathies.

## Introduction

WWOX (WW domain containing oxidoreductase) is a ubiquitously expressed tumor suppressor gene mapping to chr16q23, which spans one of the most common chromosomal fragile sites in the human genome, *FRA16D* (1–4). Tumor copy number alterations analyses revealed *WWOX* to be one of the most frequently deleted genes in cancer (5, 6) and loss of WWOX expression is characteristic of poor prognosis [Reviewed in (7)]. Although loss of WWOX is correlated with cancer development and progression, it does not behave as a highly penetrant classical tumor suppressor in most mouse models. Complete *Wwox* deletion results in post-natal death by 3–4 weeks of age (8, 9) and tissue specific deletion using a variety of Cre expression mouse models did not result in spontaneous tumor formation in mice from mixed genetic background [Reviewed in (7)]. Recently however, increased mammary carcinogenesis has been reported in a cancer susceptible mouse genetic background, supporting the hypothesis of Wwox operating as a tumor suppressor (10). We have previously observed that hypomorphic *Wwox* mice developed B cell lymphomas at old age (11) and this appears in agreement with observations indicating that heterozygous mice with only a functional *Wwox* allele (i.e., *Wwox* ^+/−^) develop an increased B cell lymphoma incidence when exposed to the carcinogen ethyl-nitrosourea (8). Thus, both of these studies (8, 11) suggest a propensity of B cells for neoplastic transformation upon Wwox deficiency.

In humans, alteration and loss of WWOX has been associated with certain B cell tumors (12–15), and most notably Multiple Myeloma (MM). In MM, the t(14;16) (q32;q23) involving *IGH* (Immunoglobulin heavy chain) and *WWOX* is a primary genetic event that results in upregulation of *MAF* and characteristic of a subgroup of high-risk MM (1, 2, 16, 17). Importantly, besides of *WWOX* participation in t(14;16), lower *WWOX* expression appears to be associated with poor MM prognosis (18–21). Loss of heterozygosity at the *WWOX* locus was shown to correlate with loss of *WWOX* expression in MM cases (21). Homozygous *WWOX* deletions are indicative of poor prognosis (22) and alteration of 16q, which includes deletion of *WWOX*, were recognized by International Myeloma Working Groups as a recurrent secondary genetic event in high-risk MM (19, 20). Furthermore, in recent analyses of data from the Myeloma Genome Project, Walker et al. identified deletion 16q23.1 affecting *WWOX*, among the most common recurrent minimal copy number changes, detected in 252 out of 1,074 (23.5%) newly diagnosed MM cases characterized by whole-exome sequencing. Thus, *WWOX* deletion is one of the most common genomic abnormalities observed in MM overall (23, 24). In addition, *WWOX* gene promoter methylation was also reported to associate with disease progression (25). Despite the overwhelming evidence suggesting a connection between WWOX loss of function and B cell neoplasia, the pathophysiological role of WWOX in B cells remains unclear.

In B cell lymphomas and MM, translocations between the *IGH* locus and oncogenes are recurrent events that drive transformation (26, 27). They are thought to be primarily generated by aberrant double strand break (DSB) formation during the antibody diversification processes of class-switch recombination (CSR) and V(D)J recombination (28, 29). Site-specific break formation at the *IGH* locus and off-target sites are determinants that impact the location, while break frequency and persistence impact the rate of translocations (30). As such, efficient DNA damage response and repair of DSBs is important in suppressing translocations (28). The major DSB pathway that operates during CSR and VDJ is the Ku70/80-dependent classical non-homologous end-joining (C-NHEJ) pathway. In the absence of C-NHEJ factors, DSBs are repaired by alternative end-joining pathways (Alt-NHEJ). These ill-defined pathways are thought to be less efficient, requiring more extensive end-processing and are biased toward microhomology based repair. In this context, studies suggest that Alt-NHEJ is prone to generating translocations while efficient repair by C-NHEJ suppresses translocations and genomic instability (28, 31). In this study, we examine the consequence of conditional B cell *Wwox* deletion in mice and find reduced survival, tumor formation, and evidence of plasma cell neoplastic transformation. Analysis of CSR from *Wwox*-deficient B cells reveals inappropriate utilization of the Alt-NHEJ pathway together with an increase in the generation of oncogenic translocations. Thus, this study demonstrates a direct role for Wwox in B cell genome stability during processes that lead to neoplastic transformation.

## Materials and Methods

### Animals

All animal research was conducted in facilities accredited by the Association for Assessment and Accreditation of Laboratory Animal Care International at the University of Texas MD Anderson Cancer Center, Science Park and all research was approved by the Institutional Animal Care and Use Committee. *BK5-Cr*e^*TG*^ and *Cd19*^*Cre*^ mice (32, 33) and the protocol to generate *Wwo*x^*flox*/*flox*^ and *Wwox knock-out (KO)* mice have been previously described (9, 34). In brief, to generate *Wwox KO* mice we crossed *Wwo*x^*flox*/*wt*^males with *BK5-Cr*e^*TG*/*TG*^*, Wwo*x^*flox*/*wt*^females (both in mixed 129SV/C57Bl/6 background). Cre expression and deletion occurs in the oocyte and embryo resulting in general recombination and deletion (33). As controls (denoted *Wwox WT) BK5-Cr*e^*TG*/0^ *Wwo*x^+/+^were used. Two-week-old *Wwox KO* mice and age matched control *Wwox WT* were compared in experimental analyses. For the generation of *Cd19 Wwox KO* we crossed *Wwo*x^*flox*/*wt*^ with *Wwo*x^*flox*/*wt*^ *Cd19*^*Cre*/+^ mice. For control mice (littermates denoted *Cd19 Wwox WT*) *Wwo*x^*wt*/*wt*^*, Cd19*^*Cre*/+^ mice were used. Mice of both genders were used in analysis. Genotypes were confirmed by PCR using primers previously described (9, 34) and see Supplementary Figure 1.

### Antibodies

The following antibodies were used: Anti-CD3 (Biolegend 100237), anti-CD11b, (Biolegend 101237), anti-IgM (Biolegend 406525), anti-CD19 (BD Biosciences 561739), anti-CD95 (BD Biosciences 561856), anti-GL7 (BD Biosciences 553666), anti-CD21 APC (BD Biosciences 561770), anti-CD138 (BD Biosciences 553714), Horseradish peroxidase conjugated anti-mouse Light Chain Specific goat polyclonal antibody (Jackson Immunoresearch #115-035-174), anti-IgG1 (BD Biosciences 560089), and for histology anti-CD45R/B220 (Bio-Rad, MCA1258G) and anti-CD138 (Biolegend 142502).

### Flow Cytometry and *Wwox* Expression Analysis in Primary Lymphocytes

The following cell populations were isolated from spleen or bone marrow by FACS sorting**:** Pro-B B220^+^/CD43^hi^/IgM^−^, Pre-B B220^+^/CD43^lo^/IgM^−^, Plasmablasts B220^+^/CD138^+^/CD19^lo^, Plasma cells B220^−^/CD138^+^/CD19^−^, Follicular B220^+^/CD19^+^/CD23^hi^/CD21^lo^, Marginal zone B220^+^/CD19^+^/Cd23^lo^/CD21^hi^, Germinal center CD19^+^/GL7^+^/CD95^+^. Viable cells were identified by forward and side scatter as well as propidium iodide dye exclusion. CD3^+^ and CD11b^+^ cells were used to exclude non-B cell populations. Samples were sorted using a BD FACSARIA Fusion. RNA from each B lymphocyte subpopulation was isolated using Trizol Reagent (Ambion) and cleaned up with RNeasy kit (Qiagen). Quantitative gene expression by reverse transcription real time PCR was determined using primer/probe Taqman set spanning exons 6–7 of *Wwox* (Thermo Fisher Scientific Assay ID Mm01247384, 4351372) and normalized to 18S RNA.

### Histology and Immunohistochemistry

Full necropsy was performed on all mice and samples from major organs and tumors (whenever available) were collected. Tissues were processed by means of formalin fixation, paraffin embedding and hematoxylin and eosin (H&E) staining. Tumor samples were also analyzed by immunohistochemistry (IHC) following standard procedures and staining with anti-CD45R/B220 and anti-CD138. Histological analyses were performed by two pathologists without prior knowledge of genotypes. Based on IHC staining pattern and cell morphology, tumors were classified following guidelines of the Bethesda classification of lymphoid neoplasms in mice and a more recent classification of mouse plasmacytomas (35, 36).

### Serum Protein Electrophoresis (SPEP)

Mice blood samples were collected at time of euthanasia. Samples were allowed to coagulate at room temperature and spun at 3000 × G for 10 min; 0.5 μl of sera were loaded in precast QuickGels (Helena Laboratories, 3505T) and run on a QuickGel Chamber (Helena Laboratories, 1284) according to the manufacturer's instructions. Samples were analyzed in triplicate with M-spike positive samples visible in all three analyses.

### Statistical Analysis for Mouse Survival and Tumor Incidence

Analyses were performed using SPSS statistical software. Log-rank test was applied to compare survival curves. Fisher's exact test was used to compare tumor incidence rates. *P*-values of < 0.05 were considered significant.

### B Cell Culture and Analysis

B cell isolation and culture have been previously described (37). Naïve B cells were purified from spleens of wild-type and *Wwox KO* 16–17 day-old mice (38, 39) by anti-CD43 bead depletion (Miltenyi Biotec). Cells were cultured in LPS (25 μg/ml, Sigma-Aldrich) and IL-4 (5 ng/ml, RD) for 72 h. To determine CSR to IgG1, cultured B cells were stained with anti-IgG1 antibodies and flow cytometric analysis of surface Ig expression was performed on a LSRFortessa (BD) with scatter gating and propidium iodide staining to exclude dead cells (40). Results were analyzed with FlowJo (Tree Star) and averages were obtained from triplicate cultures of six individual spleens in four independent experiments. Cell proliferation was analyzed with Cell Trace Violet (Invitrogen) according to manufacturer's protocol (41). Fluorescent intensity was measured at culture initiation and at 72 h. Two independent experiments were performed. The chromosome translocation assay has been previously described (42, 43). Genomic DNA was isolated from naïve B cells cultured to undergo CSR with LPS and IL-4 for 72 h. PCR with genomic DNA of 10^5^ cells per reaction was performed. 1st round PCR primers:(5′-ACTATGCTATGGACTACTGGGGTCAAG-3′ and 5′-GTGAAAACCGACTGTGGCCCTGGAA-3′) and 2^nd^ round PCR primers (5′-CCTCAGTCACCGTCTCCTCAGGTA-3′ and 5′-GTGGAGGTGTATGGGGTGTAGAC-3′) were used to amplify *Myc/Igh* translocations. Amplicons were confirmed as translocations with reactivity to both southern blot probes *Myc* (5′-GGACTGCGCAGGGAGACCTACAGGGG-3′) and Igh (5′-GAGGGAGCCGGCTGAGAGAAGTTGGG-3′). The data shown is the summary of three independent experiments and the *p*-value was calculated by two-tailed Fisher's exact test.

### Class Switch Recombination Junction Analysis

CSR junctions were amplified from genomic DNA by PCR with primers and conditions previously described (44): 36 cycles of PCR (94°C 30 s, 62°C 30 s, 68°C 8 min) using first round primers (5′-CAGGCTAAGAAGGCAATCCTGG-3′) and (5′-TTGACCTGTAACCTACCCAGGAGAC-3′); and 36 cycles of PCR (94°C 30 s, 64°C 30 s, 68°C 8 min) using second round primers (5′-GATCCAAGGTGAGTGTGAGAGGACA-3′) (5′-CATCCTGTCACCTATACAGCTAAGCTG-3′). PCR products between 0.5 and 3 Kb were purified and cloned into pCR4-TOPO (Invitrogen), individual bacterial clones were picked up and sequenced. Sequences were analyzed from three independent experiments for microhomology and mutation. To identify junctions, donor and acceptor switch regions were aligned. MH was determined by identifying the longest overlap region at the junction. A single mismatch or gap was permitted if at least 5 bp from end of the overlapping sequence in the alignment (45, 46). For mutational analysis, 100 bp from junction were analyzed on both acceptor and donor regions.

## Results

### *Wwox* Ablation in Cd19^+^ B Cells Reduces Survival and Induces Neoplastic Transformation

**Figure 1:**
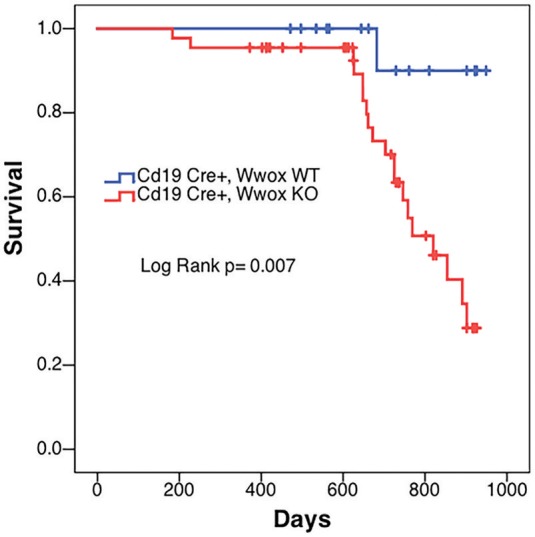
Decrease survival of *Cd19 Wwox KO* mice. Comparative survival in days of a cohort of 17 *Cd19*^*Cre*/+^ *Wwo*x^+/+^ control mice (*Cd19 Wwox WT*, blue line) vs. 44 *Cd19*^*Cre*/+^ *Wwo*x^*flox*/*flox*^ (*Cd19 Wwox KO*, red line) mice, *p*-value of Log Rank (Mantel-Cox) analysis shown. *Cd19 Wwox KO* mice had a statistically significant lower survival rate than control (*p* = 0.007). *Cd19 Wwox KO* mice displayed a mean survival of 777 vs. 922 days for control mice.

**Figure 2:**
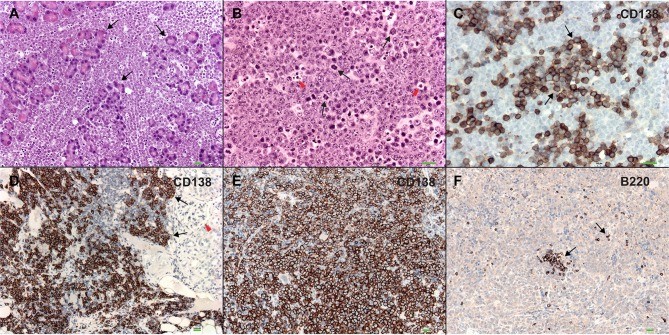
Plasmablastic plasmacytomas in *Cd19 Wwox KO* mice. **(A)** Microphotograph of plasmablastic plasmacytoma from a *Cd19 Wwox KO* mouse infiltrating pancreas, magnification 10x with H&E staining. Note how pancreatic tissue architecture is totally destroyed and replaced by infiltrating plasmacytoma cells. Black arrows point to pancreatic acinar cells. **(B)** Plasmacytoma cells infiltrating para-pancreatic lymph nodes of the same case as in **(A)**. Black arrows point to some of multiple mitoses, red arrowheads point to apoptotic bodies. Magnification 20x, H&E staining. **(C)** Anti-CD138 immunostaining of tumor shown in **(B)**. Black arrows point to CD138 positive (brown) cells. As previously described not all plasmacytoma plasmablast cells stain with anti-CD138 antibody (36). Magnification 20x, counterstained with light hematoxylin. **(D)** Heavy CD138+ cells infiltrate in kidney from another *Cd19 Wwox KO* mouse, red arrowhead points to glomerulus. **(E)** Anti-CD138 immunostaining of spleen from a different *Cd19 Wwox KO* mouse to those shown in **(A–D)**. Note that most cells are positive for the CD138+ plasma cell surface marker (brown stained cells). **(F)** Same spleen sample as in **(E)** immunostained with anti-B220 (CD45R), as can be observed very few scattered cells are positive for this B lymphocyte marker (black arrows). Both of the mice (represented in **D–F**) displayed M spikes in SPEP analyses. Microphotographs **(D–F)** taken at 10x, light hematoxylin counterstaining. Horizontal green bars at lower right corner of each photograph represent 100 μm scale.

In order to better understand the role of WWOX in B cell neoplasms we targeted deletion of this gene early in B-cell development by crossing *Wwo*x^*flox*/*flo*^^x^ mice (9) to *Cd19*^*Cre*^ transgenic mice (32). We confirmed *Wwox* protein ablation in Cd19^+^ B cells from *Cd19*^*Cre*/+^ *Wwo*x^*flox*/*flox*^ *(Cd19 Wwox KO)* mice by means of immunoblot (Supplementary Figure 1). Wwox protein was not detected in B cell samples from all analyzed *Cd19 Wwox KO* mice indicating efficient deletion in B cells (data not shown). Survival of *Cd19 Wwox KO* mice was compared with *Cd19*^*Cre*/+^ *Wwo*x^+/+^ *(Cd19 Wwox WT)* control littermates. Overall survival was plotted using the Kaplan-Meier method and analyzed by the Log-rank test. *Cd19 Wwox KO* mice had a statistically significant lower survival rate than control (*p* = 0.007, Figure 1). *Cd19 Wwox KO* mice displayed a mean survival of 777 vs. 922 days for control mice. Animals were necropsied and histology samples obtained at either the end of the experiment or when moribund. Importantly, we observed that several *Cd19 Wwox KO* mice developed intra-abdominal tumors with the characteristics of B cell neoplasms (Figure 2). Tumors were broadly classified into lymphomas and plasmacytomas based on the IHC staining pattern (Table 1). Neoplastic lesions that were exclusively B220+ indicative of B cell lymphomas were further classified into mature and immature lymphomas based on published guidelines (35). Several of the observed intra-abdominal tumors were positive for B220 and CD138, suggestive of plasmablastic plasmacytomas predominantly affecting mesenteric and retroperitoneal lymph nodes, and were histologically classified as previously described (36) (Figure 2 and Table 1). Tumors were also tested for Wwox immunoreactivity and were negative (not shown). Of 34 *Cd19 Wwox KO* mice, 8 (23.4%) developed (B220^+^) B cell lymphomas and 8 other mice developed plasmacytomas (B220^+^,CD138^+^) predominantly affecting mesenteric and retroperitoneal lymph nodes. Thus, a total of 16 of 34 (47%) *Cd19 Wwox KO* mice developed B cell neoplasms. In the *Cd19 Wwox WT* control group, only 2 of 14 (14.3%) mice developed B cell lymphomas and none developed plasmacytomas. In summary, the B-cell tumor incidence rates between the *Cd19 Wwox KO* group and the *Cd19 Wwox WT* group were significantly different (*p* < 0.05).

**Table 1:** Histopathology of detected tumors and status of SPEP results.

**Mouse ID**	**Genotype [Cd19 Wwox]**	**Age (ds)**	**Histopathology**	**M SPIKE in SPEP**
15	KO	735	Mature B cell lymphoma	**+**
16	KO	735	Mature B cell lymphoma	n/d
68	KO	648	Plasmablastic plasmacytoma (in MLN)	–
70	KO	648	Plasmablastic plasmacytoma (in MLN)	**+**
93	KO	827	Anaplastic plasmacytoma	**+**
95	KO	820	Anaplastic plasmacytoma	**+**
97	KO	820	Plasmacytoma (in MLN)	**+**
103	KO	626	Anaplastic plasmacytoma	–
104	KO	625	Precursor B cell lymphoma	n/d
161	KO	602	Precursor B cell lymphoma	n/d
189	KO	769	Precursor B cell lymphoma	**+**
231	KO	737	Mature B cell lymphoma	–
254	KO	725	Precursor B cell lymphoma	–
261	KO	746	Plasmablastic plasmacytoma (in MLN)	n/d
269	KO	717	No macroscopic tumor	**+**
312	KO	921	Mature B cell lymphoma	**+**
332	KO	917	No macroscopic tumor	**+**
334	KO	891	Anaplastic plasmacytoma	**+**
180	WT	729	No macroscopic tumor	**+**
295	WT	926	Mature B cell lymphoma	–
325	WT	922	Mature B cell lymphoma	n/d

*MLN, Mesenteric Lymph Node; + indicates positive for M spikes; − indicates negative for M spikes as per SPEP analysis; n/d, not determined*.

**Figure 3:**
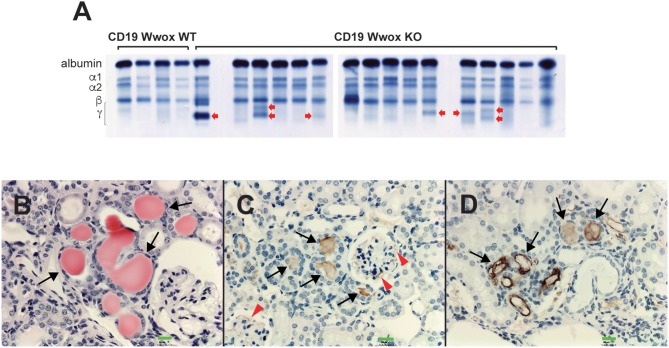
SPEP gels and MM cast nephropathy. *Cd19 Wwox KO* mice displayed evidence of monoclonal gammopathies. **(A)** Representative SPEP of serum samples from *CD19 Wwox KO* and *WT* control mice. Red arrows point to some representative M spikes. All samples were analyzed in triplicate. Ten of 27 (37%) *Cd19 Wwox KO* mice displayed detectable M-spikes vs. only 1 of 9 (11%) tested *Cd19 Wwox WT* control mice. **(B–D)** Myeloma Cast Nephropathy. H&E staining and anti-mouse IgG of kidney tissue sections from a *Cd19 Wwox KO* displaying an M spike and poor health at 820 days of age. **(B)** Black arrows indicate eosinophilic (protein) deposits obstructing kidney tubules. **(C,D)** same kidney sample as shown in **(B)** immunostained using a goat polyclonal anti-mouse IgG, light chain-specific peroxidase conjugated antibody, counterstaining with light hematoxylin. Brown staining of the intratubular protein deposits are formed by Ig light chain aggregates (black arrows). Ig light chain deposits can also be observed in the mesangial area of glomeruli [red arrowheads in **(C)**]. All microphotographs taken at 20x magnification, 100 μM scale bars are shown.

A characteristic of multiple myeloma, plasmacytomas and other plasma cell neoplasms is the secretion of immunoglobulin (Ig), detected in serum protein electrophoresis (SPEP) as Ig monoclonal bands (M-spikes) which is a routine method used in the clinic for the diagnosis of plasma cell dyscrasias. Serum samples were obtained from morbid mice prior to euthanasia whenever possible and those that survived to the end of the experimental period. SPEP analyses revealed M-spikes in multiple *Cd19 Wwox KO* mice (Figure 3A). In total 10 of 27 (37%) *Cd19 Wwox KO* mice but only 1 of 9 (11%) tested *Cd19 Wwox WT* control mice had detectable M-spikes. Full histopathologic analyses also indicated that some of the mice with M-spikes displayed evidence of myeloma cast nephropathy (myeloma kidney). This is characterized by the presence of obstructing casts in the lumen of kidney tubules. These eosinophilic (pink) deposits are formed by aggregates of monoclonal Ig light chains (Figures 3B–D).

### Wwox Is Expressed in B Cells and Functions During Ig Class Switching

**Figure 4:**
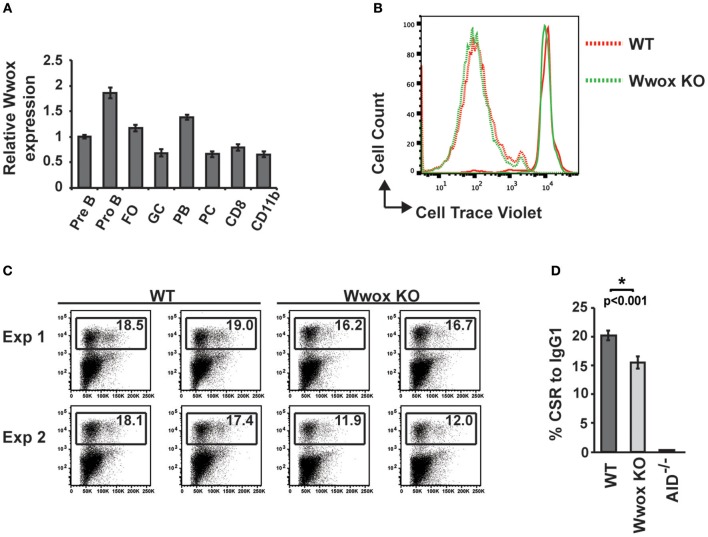
Wwox B cell expression and function during Ig class-switching. **(A)** Wwox transcript level analysis at various B cell differentiation stages. Pre B (B220^+^/CD43^lo^/IgM^−^), Pro B (B220^+^/CD43^hi^/IgM^−^), follicular ([FO], B220^+^/CD19^+^/CD23^hi^/CD21^lo^), germinal center ([GC], CD19^+^/GL7^+^/CD95^+^), Plasmablast ([PB], B220^+^/CD138^+^/CD19^lo^), and Plasma cell ([PC], B220^−^/CD138^+^/CD19^−^) B cells; CD8^+^ T cells; and CD11b^+^ populations were FACS sorted from the spleen or bone marrow of unimmunized wild-type mice. Relative levels of Wwox were assessed by RT-qPCR (Taqman assay) and normalized to 18S RNA levels. Results shown are mean values of triplicate analyses (error bars SEM). **(B)** Representative histogram of B cells proliferation analysis. Fluorescent intensity of baseline labeling (solid lines) of naïve B cells isolated from *Wwox WT* or *Wwox KO* spleens and after 72 h culture with IL4 and LPS (dotted lines). **(C)** Representative flow cytometry plots of CSR to IgG1 after 72 h culture. Relative percentage of cells expressing IgG1 is indicated on each plot. Duplicate plots from two experiments displayed. **(D)** Mean values of CSR to IgG1 in activated B cells from *n* = 4 independent experiments, (error bars SEM). *P* value was determined by a two-tailed *t*-test assuming unequal variance, ^*^ indicates significant *p* value.

To address when during B cell development Wwox may be suppressing tumorigenesis, we first assessed normal *Wwox* expression in various B cell compartments. B cells from Pro-B, Pre-B, marginal zone, follicular B, germinal center (GC), plasmablast, and plasma cell compartments were purified from the spleen or bone marrow of wild-type mice. *Wwox* expression was analyzed by qPCR and compared to that of splenic CD8^+^ lymphocytes and CD11b^+^ leukocytes (Figure 4A). We find *Wwox* expression throughout B cell development including germinal center cells where *Ig* genes undergo mutagenic rearrangement. During *Igh* CSR, the enzyme AID (activation-induced cytidine deaminase) induces DSB in the switch regions, a process that is a significant source of genomic instability. To determine if Wwox functions during CSR we analyzed B cells for the ability to undergo proper CSR. Naïve (IgM^+^) B cells were isolated from the spleens of wild-type, *Wwox KO* and *Wwo*x^+/−^ 15 days old mice. Cells were cultured with LPS and IL-4 for 72 h to stimulate proliferation and CSR to *IgG1*. Cell proliferation was normal and flow cytometric analysis of cell surface antibody isotype expression revealed that *Wwox KO* cells supported *IgG1* CSR at a rate of ~75% of wild-type controls (Figures 4B–D).

### Wwox Deficiency Impairs C-NHEJ and Promotes Alt-NHEJ During Ig Class Switching

**Figure 5:**
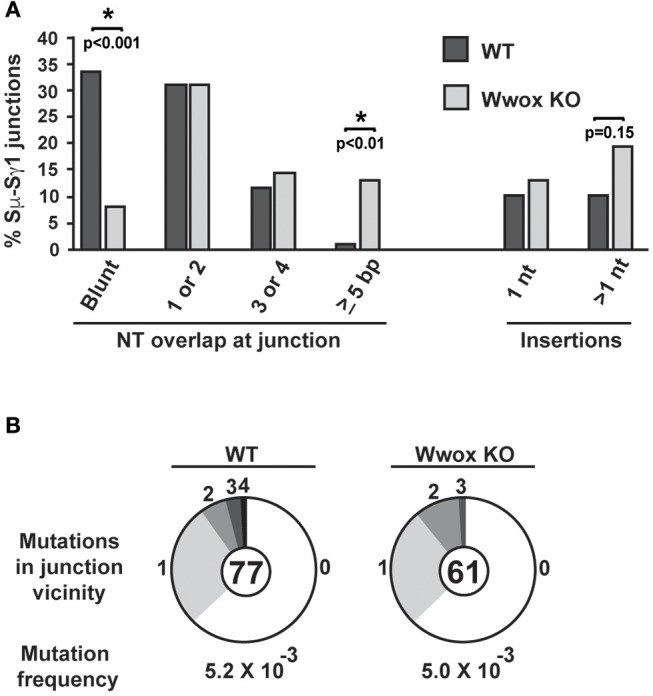
Analysis of class switch recombination junctions. **(A)** Naïve B cells isolated from the spleens of *Wwox WT* or *Wwox KO* mice were induced to undergo CSR to IgG1 with IL4 and LPS for 72 h. Genomic DNA was isolated, Sμ-Sγ junctions were PCR amplified, cloned and sequenced. The percentage of junctions with the denoted number of nucleotide (NT) overlap or insertion is indicated (a total of 76 WT and 61 *Wwox KO* unique clones, from *n* = 4 experiments were analyzed, see also Supplementary Figure 2). A significant decrease in blunt end joins can be observed in *WWOX KO* B cells (*Wwox WT*, 26 blunt of 77 total junctions [i.e., 33.8%] vs. *Wwox KO*, 5 blunt [8.2%] of 61 total junctions analyzed). Long MHs (>5 bp MH) were rare in *WT* B cells but significantly increased in *Wwox KO* B cells (*Wwox WT*, 1 of 77 [1.3%] vs. *Wwox KO*, 8 of 61 [13.1%]). *P* values were determined using two-tailed Fisher's exact test, ^*^ indicates significant *p* value. **(B)** Mutations within the vicinity of CSR junctions. 100 bp flanking the 5′ and the 3′ of the Sμ-Sγ breakpoint were analyzed for mutations. Segment sizes in the pie charts are the proportion of clones that contained the number of mutations indicated in the periphery of the charts. The total number of independent junctions analyzed is indicated in the center of each chart. Analysis of mutation frequency within 100 bp of the CSR junction revealed no difference between *Wwox WT* and *Wwox KO* cells (5.2 × 10^−3^ vs. 5.0 × 10^−3^ mutations/bp, respectively, *p* = 0.8 two-tailed *t*-test, assuming unequal variance). Frequency denoted as mutations per base pair.

CSR requires end-joining between DSBs to exchange one isotype constant region for another. DSBs are induced and resolved during phase G1 of the cell cycle predominantly by the C-NHEJ pathway. In the absence of C-NHEJ, CSR is supported at a slightly reduced rate by alternative end-joining activity (Alt-NHEJ), with recombination junctions biased toward microhomology-mediated (MH) end-joining (MMEJ) (38, 45–49). To determine if Wwox deficiency altered C-NHEJ engagement we analyzed CSR junctions for MH usage following standard methods (44–49). To this end, the Sμ-Sγ1 junctions were amplified, PCR products were purified, cloned and sequenced from pools of B cells induced to undergo CSR to *IgG*. Sequence analysis of individual clones representative of both conditions (i.e., *Wwox WT* vs. *Wwox KO* B cells) revealed significant differences in the overall use of donor/acceptor homology at the CSR junctions (two-tailed Mann-Whitney test, *p* < 0.001). *Wwox*-deficient B cells displayed a significant shift toward microhomology (average MH *Wwox WT* 1.2 bp vs. *Wwox KO* 2.6 bp). Importantly, there was a significant decrease in blunt end joins (*Wwox WT*, 26 blunt of 77 total junctions [i.e., 33.8%] vs. *Wwox KO*, 5 blunt [8.2%] of 61 total junctions analyzed, *p* < 0.001 Fisher's exact test). Long MHs (>5 bp MH) were rare in *WT* B cells but significantly increased in *Wwox KO* B cells (*Wwox WT*, 1 of 77 [1.3%] vs. *Wwox KO*, 8 of 61 [13.1%], *p* < 0.01 Fisher's exact test) (Figure 5A and Supplementary Figure 2). The overall frequency of nucleotide insertions at CSR junctions was not significantly different, although *Wwox KO* B cells displayed a slight increase of insertions >1 bp (Figure 5A). Mutations occur in the vicinity of the junctions and are thought to be due to the AID induced mutational processes engaged during CSR (50). Analysis of mutation frequency within 100 bp of the CSR junction revealed no difference between *Wwox WT* and *Wwox KO* (5.2 × 10^−3^ vs. 5.0 × 10^−3^ mutations/bp respectively, *p* = 0.8 *t*-test) (Figure 5B). We conclude that Wwox deficiency results in a significant shift toward MMEJ between switch regions, consistent with a shift toward the Alt-NHEJ pathway in the repair of AID induced DSBs.

### Increased Translocations in *Wwox KO* B Cells Undergoing Class Switch Recombination

**Figure 6:**
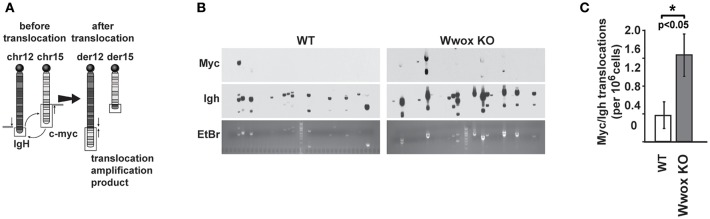
Wwox-deficient B cells display increased frequency of spontaneous *Myc/Igh* translocations. **(A)** Schematic for the *Myc*/*Igh* translocation assay. PCR amplification primers for mouse chromosomes 12 (*Igh* locus) and 15 (*Myc* locus) are represented by black arrows and Southern probes by gray bars. Naïve B cells from 15 day old *Wwox WT* or *Wwox KO* mice were cultured with LPS and IL-4 for 72 h and assayed for translocations. **(B)** Representative translocation detection assay performed by means of Southern blots using *Myc* and *Igh* probes and corresponding ethidium bromide stained gels. Each lane represents the amplification result from 1 × 10^5^ cells. **(C)** Summary of translocation events detected. Total frequency from *n* = 4 independent experiments. *P* value determined using two-tailed Fisher's exact test, ^*^indicates significant *p* value.

Although AID preferentially targets *Ig* genes, off-target AID activity can induce DSBs at other sites providing recombination substrates that result in characteristic chromosome translocations frequently found in B cell tumors (51, 52). One example is the oncogenic *MYC/IGH* translocation found in Burkitt's lymphoma and other B cell tumors. This translocation is mediated by AID induced DSBs at both *MYC* and *IGH*, with breaks in the *MYC* gene being rate-limiting (43, 53). Chromosome translocations display signatures of Alt-NHEJ repair in B cells while C-NHEJ suppresses translocation formation (47). To determine if *Wwox* had a role in the incidence of spontaneous chromosome translocations during CSR, we used a previously described PCR/Southern blot assay to measure the frequency of AID induced *Myc/Igh* translocations (43, 53) (Figure 6A). It has been shown that naïve B cells do not harbor *Myc/Igh* translocations while cells induced to undergo CSR display translocations in an AID dependent fashion (43, 54). We observed that compared to *Wwox WT, Wwox-*deficient B cells had a significant 2.5-fold increase in spontaneous translocations (Figures 6B,C). Thus, we find that lack of Wwox expression is affecting the generation of spontaneous chromosome translocations, a process known to be promoted by the Alt-NHEJ pathway (47).

## Discussion

The incidence of plasma cell dyscrasias observed in our mouse model demonstrates that ablation of *Wwox* contributes to disease. Several mechanisms can mediate the loss of WWOX expression in human plasma cell dyscrasias including promoter methylation (25), genomic deletions (18, 21–24) and translocations (16, 17). One translocation example, t(14;16), is known as a MM high-risk indicator (20). The chromosome 16 breakpoints of t(14;16) are found within the *WWOX* gene and as a consequence structurally disruptive of the affected allele. The *MAF* locus, located 3' of *WWOX*, is brought within the influence of chromosome 14 *IGH* control elements and the resulting MAF overexpression has a causative role in MM. It is unclear however whether *WWOX* haploinsufficiency plays any role, or if silencing of the remaining allele via additional structural disruption (e.g., deletion) or epigenetic mechanisms is required in order to contribute to disease progression.

It has been shown that *Wwox* does not behave like a highly penetrant tumor suppressor gene [Reviewed in (7)]. The limited tumor incidence, long latency and heterogeneity of neoplastic transformation in our mouse model suggests that additional secondary genetic events have to occur for full-blown malignancy to develop. Nevertheless, the lack of tumorigenesis when *Wwox* is conditionally deleted in various other tissues in mice with the same mixed genetic background as those here used (7) together with previous observations (8, 11), suggests that B cells are indeed a target tissue in which *Wwox* ablation contributes to neoplastic transformation.

Lymphocytes are unique in that they undergo programmed DNA damage during their receptor diversification processes. While T and B cells undergo V(D)J recombination only B cells undergo CSR and somatic hypermutation. The genomic instability induced by these B cell specific processes likely drives the imbalance of 95% of lymphomas being B cell derived with the majority being of post-germinal center origin (26). During CSR, activation-induced cytidine deaminase (AID) induces DSB in the *IGH* switch regions to trigger recombination between isotype constant regions. The generation and resolution of these breaks occurs during the G1 phase of the cell cycle when NHEJ repair, but not homologous recombination, is readily available. Breaks randomly occur throughout each isotype switch region which are sequence diverse from each other. The result generates incompatible DSB end structures that require end-processing for ligation. The Ku70/80-mediated C-NHEJ pathway is the major pathway engaged during CSR. Recombination junctions normally lack microhomology consistent with the ability of C-NHEJ factors including XRCC4-DNA Ligase IV (Lig4) to process non-compatible ends (47). If C-NHEJ is unavailable, CSR proceeds with reduced efficiency via Alt-NHEJ, which tends to utilize MMEJ (46). We find that *Wwox*-deficient B cells support CSR at a slightly reduced rate and display MMEJ at recombination junctions consistent with Alt-NHEJ repair (46). MMEJ is always mutagenic since extensive end resection occurs to find microhomologous regions with repair deleting some intervening sequence (55). Alt-NHEJ has been implicated in chromosome translocations, as junction MH is a frequent feature of breakpoints and loss of C-NHEJ activity increases translocations (47, 56). For example, *Ku70* or *Lig4*-deficient B cells generate frequent *Myc/Igh* translocations via an Alt-NHEJ joining mechanism (49). Therefore, inappropriate usage of the Alt-NHEJ pathway has the potential to destabilize the genome. In this study, we find that *Wwox* deficiency increased generation of spontaneous B cell *Myc/Igh* translocations. This translocation is generated by AID induced DSBs at both Myc and *Igh* in primary B cells undergoing CSR (43, 53). Such translocation is a primary event in lymphomas such as Burkitt's and a common MM secondary event. Translocation capture assays show *Myc/Igh* to be indicative of genome wide *Igh* translocation and so determination of the spontaneous occurrence of such translocation event serves as a surrogate marker for overall genome instability (51, 52). In *Wwox* deficient B cells AID levels are not higher than WT (Supplementary Figure 3) and mutation frequency in the vicinity of recombination junctions is not increased. This further supports that *Wwox* deficiency influences DSB repair and not the mutation-generating machinery. Although AID is not expressed in MM cell lines, interaction of MM with dendritic cells in the *in vivo* microenvironment provides conditions that could induce AID expression and genomic instability (57). Furthermore, mutational signatures from APOBEC deaminase family members are found in MM and are associated with poor prognosis (58). Therefore, a primary event involving loss of WWOX could influence genome stability during periodic AID or APOBEC expression in MM.

The mechanism by which Wwox influences C-NHEJ and Alt-NHEJ during B cell CSR is not clear. Previous studies have indicated that *WWOX* deficiency in human cell lines results in genome instability and abnormalities in DNA damage repair pathways (59, 60). Abu-Odeh et al. have reported that WWOX depletion can lead to reduced ATM checkpoint kinase activation and impaired DNA repair (59). In support of the observations here described, we have previously reported that WWOX depletion decreased C-NHEJ efficiency in human cell lines and is associated to the generation of phenotypes displaying increased resistance to the effects of DNA damaging agents (60, 61). However, *WWOX* function in B cells had not been previously examined. Since the DNA damage response is often dysregulated in cell lines, we here analyzed Wwox deficiency in primary B cells both in culture and *in vivo*. These results not only provide a clear and novel role for Wwox in B cell transformation and plasma cell dyscrasias but also further strengthen the notion of WWOX as an important player in maintaining genome integrity.

## Data Availability

All datasets generated for this study are included in the manuscript and/or the Supplementary Files.

## Ethics Statement

All animal research was conducted in facilities accredited by the Association for Assessment and Accreditation of Laboratory Animal Care International at the University of Texas MD Anderson Cancer Center, Science Park and all research was approved by the Institutional Animal Care and Use Committee.

## Author Contributions

CA and KM designed research. HK, YM, JP, JL, and MZ performed research. MS and CA performed pathology analyses. CA, MA, and KM analyzed the data. CA and KM wrote the paper.

### Conflict of Interest Statement

The authors declare that the research was conducted in the absence of any commercial or financial relationships that could be construed as a potential conflict of interest.

## Supplementary Material

Supplementary Figure 1 Mouse genotyping and Wwox expression ablation in B cells from *Cd19 Cre*+*, Wwo*x^*flox*/*flox*^ mice. **(A)** PCR genotype analysis of DNA from two *Cd19*^*Cre*/+^*;Wwo*x^*wt*/*flox*^, two *Cd19*^*Cre*/+^*;Wwo*x^*wt*/*wt*^, and two *Cd19* ^*Cre*/+^*;Wwo*x^*flox*/*flox*^ mice as indicated. Genotyping was performed using Cre primers F; 5′ GCC TGC ATT ACC GGT CGA TGC AAC G 3′ and R; 5′ GTG GCA GAT GGC GCG GCA ACA CCA T 3′ generating a PCR product of 700 bp in size. For amplifying the *Wwox wt* and *Wwox floxed* loci we used primers *Wwox*-N1; 5′ ATG GGA CGA AAC TGG AGC TCA GAA 3′, *Wwox*-N2; 5′ TCA GCA ACT CAC TCT GGC TTC AAC 3′ and *Wwox*-L; 5′ GCA TAC ATT ATA CGA AGT TAT TCG AG 3′, as previously described (9). The *Wwox wt* amplicon generates a 463 bp PCR product while the *Wwox* floxed allele generates a 344 bp product as indicated. **(B)** Representative immunoblot using Wwox antibody on protein extracts from FACS isolated Cd19+ B cells (first and fifth lanes) and from various other tissues (cerebellum, lung and kidney) from a *Cd19* ^*Cre*/+^*;Wwo*x^*flox*/*flox*^ *(Cd19 Wwox KO)* mouse and a *Cd19*^*Cre*/+^*;Wwo*x^*wt*/*wt*^ *(Cd19 Wwox WT)* counterpart. As can be observed Wwox protein ablation is exclusive of B cells in the *Cd19 Wwox KO* mouse with other tissues expressing normal Wwox protein levels. Loading control using Actin antibody, lower panel.

Supplementary Figure 2 Summary and alignments of CSR junctions. Summary table of number of clones with each type of junction. Representative alignments of a blunt, a 1 bp, a 2 bp, and all CSR junctions with more than 4 bp microhomology (MH). Sequence of a CSR junction (Blue, **Middle**) is aligned with germline switch donor **(Top)** and acceptor **(Bottom)** regions. Vertical lines denote identity between germline switch region and the sequenced CSR junction, bold lines mark continuous identity used to identify the breakpoint. Red denotes overlap between switch donor and acceptor region.

Supplementary Figure 3 AID levels are not altered in *Wwox KO* B cells. Mouse splenocytes from wild-type (WT), *AID KO* (*Aicda*), or *Wwox KO* mice were cultured in LPS and IL-4 for 3 days. Anti-AID (Cell Signaling Technology L7E7) and anti-tubulin (Sigma) Western blot was performed on cell lysates and the relative signal ratio of AID to tubulin is displayed. Experiment representative of 3 independent experiments.
