# Supplementary figures and images for: Wwox Deletion in Mouse B Cells Leads to Genomic Instability, Neoplastic Transformation, and Monoclonal Gammopathies
Source: Front Oncol. 2019 Jun 19;9:517. doi: 10.3389/fonc.2019.00517 (PMC6593956; doi:10.3389/fonc.2019.00517)

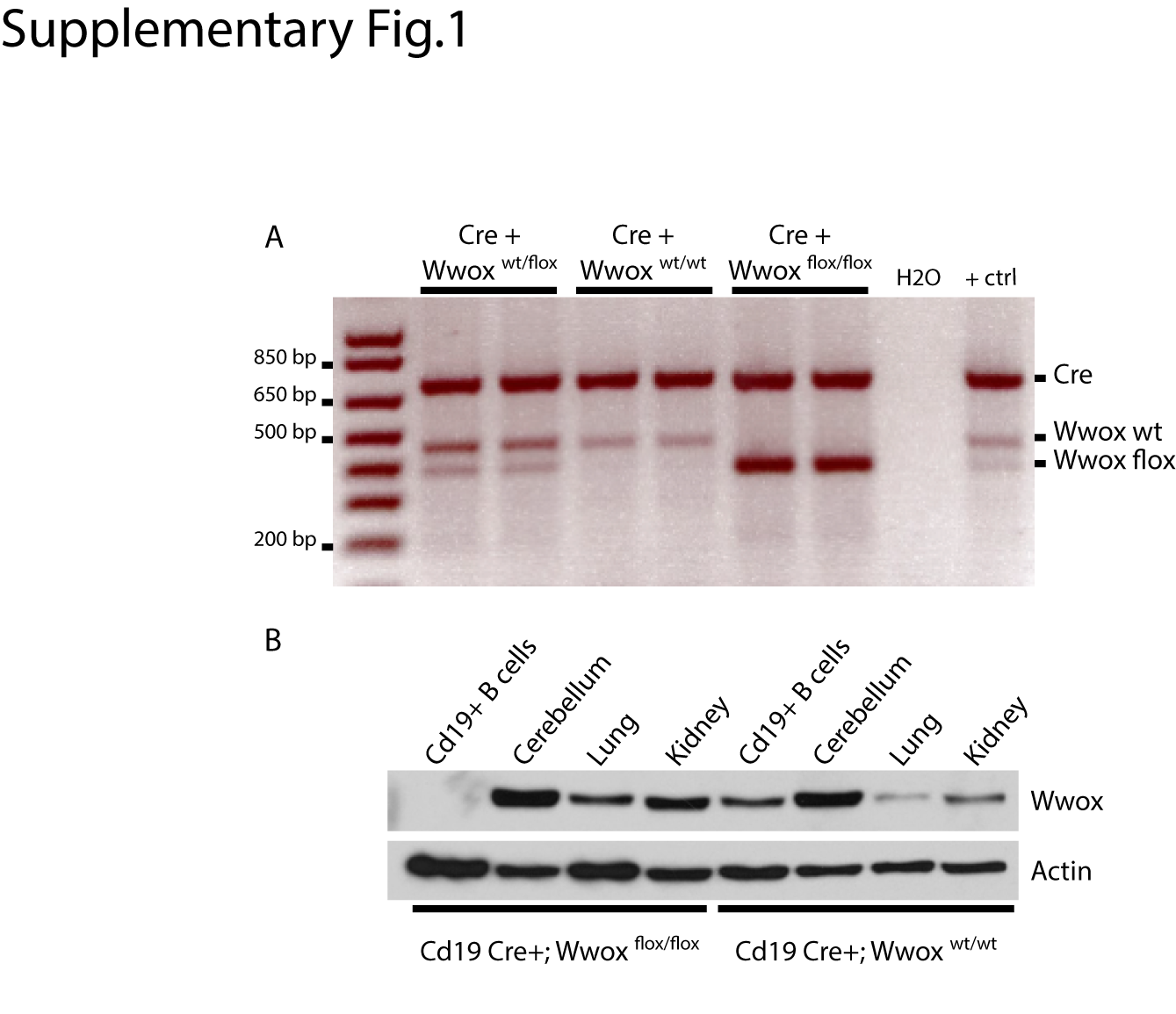

Supplement: Supplementary Figure 1 — Mouse genotyping and Wwox expression ablation in B cells from Cd19 Cre+, Wwoxflox/flox mice. (A) PCR genotype analysis of DNA from two Cd19Cre/+;Wwoxwt/flox, two Cd19Cre/+;Wwoxwt/wt, and two Cd19 Cre/+;Wwoxflox/flox mice as indicated. Genotyping was performed using Cre primers F; 5′ GCC TGC ATT ACC GGT CGA TGC AAC G 3′ and R; 5′ GTG GCA GAT GGC GCG GCA ACA CCA T 3′ generating a PCR product of 700 bp in size. For amplifying the Wwox wt and Wwox floxed loci we used primers Wwox-N1; 5′ ATG GGA CGA AAC TGG AGC TCA GAA 3′, Wwox-N2; 5′ TCA GCA ACT CAC TCT GGC TTC AAC 3′ and Wwox-L; 5′ GCA TAC ATT ATA CGA AGT TAT TCG AG 3′, as previously described (9). The Wwox wt amplicon generates a 463 bp PCR product while the Wwox floxed allele generates a 344 bp product as indicated. (B) Representative immunoblot using Wwox antibody on protein extracts from FACS isolated Cd19+ B cells (first and fifth lanes) and from various other tissues (cerebellum, lung and kidney) from a Cd19 Cre/+;Wwoxflox/flox (Cd19 Wwox KO) mouse and a Cd19Cre/+;Wwoxwt/wt (Cd19 Wwox WT) counterpart. As can be observed Wwox protein ablation is exclusive of B cells in the Cd19 Wwox KO mouse with other tissues expressing normal Wwox protein levels. Loading control using Actin antibody, lower panel. [file Image_1.TIF]

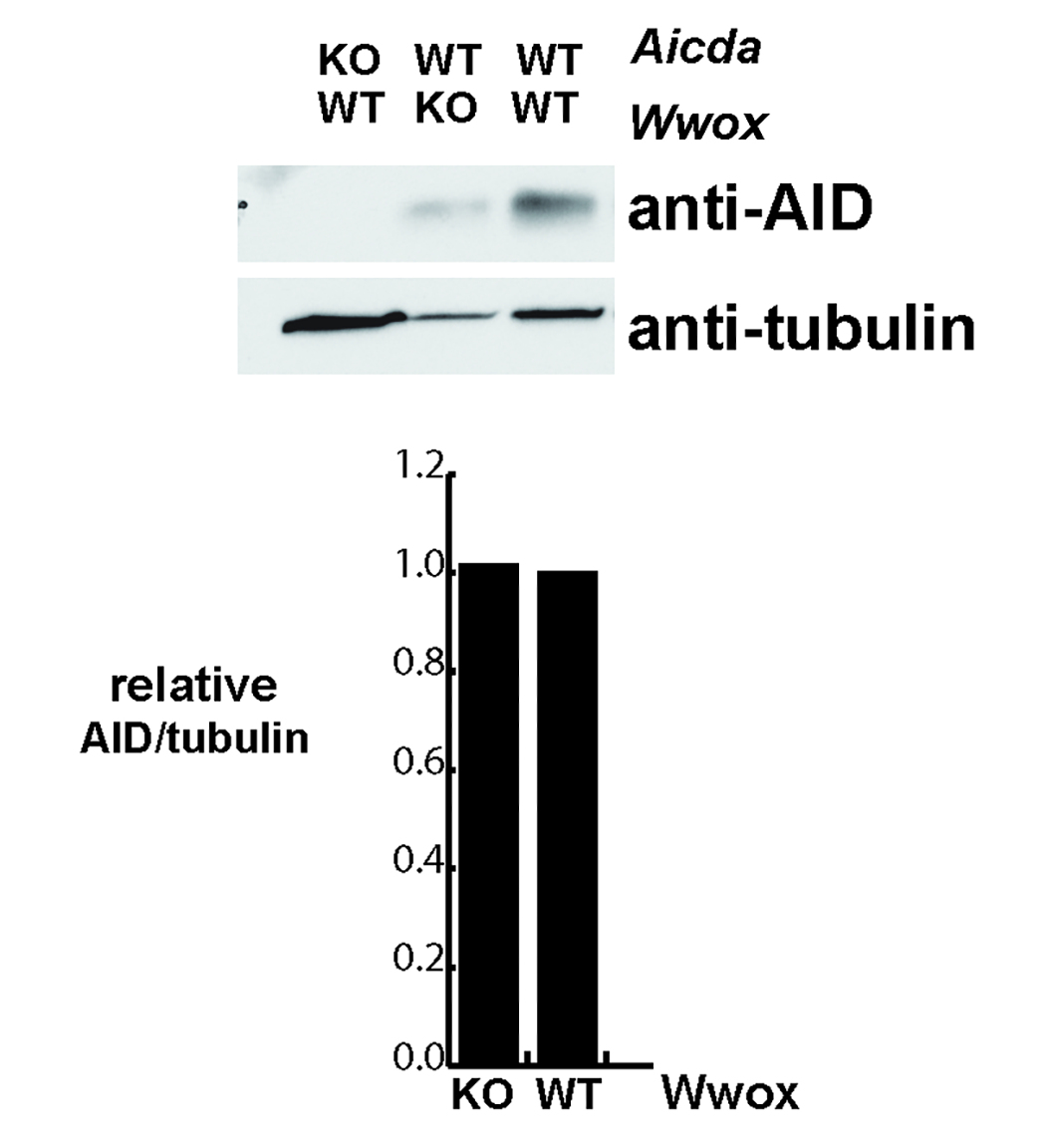

Supplement: Supplementary Figure 3 — AID levels are not altered in Wwox KO B cells. Mouse splenocytes from wild-type (WT), AID KO (Aicda), or Wwox KO mice were cultured in LPS and IL-4 for 3 days. Anti-AID (Cell Signaling Technology L7E7) and anti-tubulin (Sigma) Western blot was performed on cell lysates and the relative signal ratio of AID to tubulin is displayed. Experiment representative of 3 independent experiments. [file Image_3.tif]
