# Supplementary material for: Wwox Deletion in Mouse B Cells Leads to Genomic Instability, Neoplastic Transformation, and Monoclonal Gammopathies
Source: Front Oncol. 2019 Jun 19;9:517. doi: 10.3389/fonc.2019.00517 (PMC6593956; doi:10.3389/fonc.2019.00517)
Supplement: Supplementary Figure 2 — Summary and alignments of CSR junctions. Summary table of number of clones with each type of junction. Representative alignments of a blunt, a 1 bp, a 2 bp, and all CSR junctions with more than 4 bp microhomology (MH). Sequence of a CSR junction (Blue, Middle) is aligned with germline switch donor (Top) and acceptor (Bottom) regions. Vertical lines denote identity between germline switch region and the sequenced CSR junction, bold lines mark continuous identity used to identify the breakpoint. Red denotes overlap between switch donor and acceptor region. [file Image_2.pdf]

S2 Figure

Summary of Junction classification

**Overlap (bp)**	WT	KO
blunt	26	5
one or two	24	19
three or four	9	9
five or six	0	5
seven or eight	1	3
**Insertions**		
1 nucleotide	8	8
>1 nucleotide	9	12
**Total**	77	61

Example alignments:

Blunt

```
AGTAGACTGTAATGAACTGGAATGAGCTGGGCCGCTAAGCTAAACTAGGC    : Donor sequence (IgM germline)
|||||||||||||||||||||||||   |     |        | |
AGTAGACTGTAATGAACTGGAATGAAGGGATAGACATGTAAGCAGTCAAG    : CSR junction
    |      |   ||        |||||||||||||||||||||||||
CCAGGCTGAGCAGCTACCAAGGATCAGGGATAGACATGTAAGCAGTCAAG    : Acceptor sequence (IgG1 germline)
```

1 bp overlap

```
GGGGTATGGATACGCAGAAGGAAGGCCACAGCTGTACAGAATTGAGAAAGA
||||||||||||||||||||||||||           |  | |    |
GGGGTATGGATACGCAGAAGGAAGGCACAGATGAGCAAATACTACATAGCT
   |           || | |  | ||||| ||||||||||||||||||||
ACTGATCCAGGTGAGAGTACGGGGTCACAGCTGAGCAAATACTACATAGCT
```

2 bp overlap

```
GAGGTGATTACTCTGAGGTAAGCAAAGCTGGGCTTGAGCCAAAATGAAGTAG
|||||||||||||||||||||||||||     ||   || |    ||
GAGGTGATTACTCTGAGGTAAGCAAAGAGAAACTGAGGCAAGTGGGAGTGCA
|  |    | |  |   ||      |||||||||||||||||||||||||||
GGTGGAGGTCCAGTTGAGTGTCTTTAGAGAAACTGAGGCAAGTGGGAGTGCA
```

WWOX-KO: >4bp overlap (8 of 61 junctions)

KO - 5bp overlap

```
AGCTGAGCTTGACTGAGCTAGGGTGAGCTGGACTGAGCTGGGGTGAGCTGAGCTG
||||||||||||||||||||||||||||||  |  |       |     ||||||
AGCTGAGCTTGACTGAGCTAGGGTGAGCTG-GCAAATACTACATAGCTGGAGCTG
| |   |   || |  |        ||||| ||||||||||||||||||||||||
ATCCAGGTGAGAGTACGGGGTACACAGCTGAGCAAATACTACATAGCTGGAGCTG
```

KO - 5bp overlap

```
ACTCTGAGGTAAGCAAAGCTGGGCTTGAGCCAAAATGAAGTAGACTGTAATGAAC
||||||||||||||||||||||||||||||  |  |||  | |  ||   |||
ACTCTGAGGTAAGCAAAGCTGGGCTTGAGCTGAGCTGAGCTGGGGTGAGCTGAGT
        |            ||  ||||||||||||||||||||||||||||||
TGAGCTGAGCTGAGCTGAGCTGGGGTGAGCTGAGCTGAGCTGGGGTGAGCTGAGT
```

KO - 5bp overlap

```
AAGTAGACTGTAATGAACTGGAATGAGCTGGGCCGCTAAGCTAAACTAGGCTGGC
||||||||||||||||||||||||||||||            |      |  ||
AAGTAGACTGTAATGAACTGGAATGAGCTGAAGGTAATCTGGAGCTAGTGGGGGT
  ||  |         |     |  ||||||||||||||||||||||||||||||
GGGTGTATAAGGTACCAGGCTGAGCAGCTGAAGGTAATCTGGAGCTAGTGGGGGT
```

KO - 5bp overlap

```
CACTGGACTGTTCTGAGCTGAGATGAGCTGGGGTGAGCTCAGCTATGCTACGCTGT
||||||||||||||||||||||||||||||  |  |     |   |  |  |
CACTGGACTGTTCTGAGCTGAGATGAGCTGAAGGTAATCTGGAGCTAGTGGGGGT
   || |              ||  |||||||||||| |||||||||||||||||
GGGTGTATAAGGTACCAGGCTGAGCAGCTGAAGGTAACCTGGAGCTAGTGGGGGT
```

KO - 6bp overlap

```
ACTCTACTGCCTACACTGGACTGTTCTGAGCTGAGATGAGCTGGGGTGAGCTCAG
||||||||||||||||||||||||||||||| |        |      |     |
ACTCTACTGCCTACACTGGACTGTTCTGAGCAGCTCCAGCTTAGCTGTATAGGTG
 |      |  | ||  |  |    ||||||||||||||||||||||||||||||
GCAAGTGGGAGTGCAGAGATCCAAGCTGAGCAGCTCCAGCTTAGCTGTATAGGTG
```

KO - 7bp overlap

```
ACACTGGACTGTTCTGAGCTGAGATGAGCTGGGGTGAGCTCAGCTATGCTACGCTGT
||||||||||||||||||||||||||||||||  |||     |   | |      |
ACACTGGACTGTTCTGAGCTGAGATGAGCTGGTATGAAGGTGGAGGTCCAGTTGAGT
|  ||   ||  |    |       ||||| ||||||||||||||||||||||||||
AAGCTCAGCTACTACATGAGAGCTGGAGCTAGTATGAAGGTGGAGGTCCAGTTGAGT
```

KO - 7bp overlap

```
GAGAAGGCCAGACTCATAAAGCTTGCTGAGCAAAATTAAGGGAACAAGGTTGAGAGC
|||||||||||||||||||||||| |||||||     | |  | |  |    || |
GAGAAGGCCAGACTCGTAAAACTT-CTGAGCAGCTGAAGGTAATCTGGAGCTAGTGG
|   | |   |  |     | |   ||||||||||||||||||||||||||||||||
GCTGATGGGTGTATAAGGTACCAGGCTGAGCAGCTGAAGGTAATCTGGAGCTAGTGG
```

KO - 8bp overlap

```
GGGTATGGATACGCAGAAGGAAGGCCACAGCTGTACAGAATTGAGAAAGAATAGAGAC
|||||||||||||||||||||||||||||||||  || |    | | ||
GGGTATGGATACGCAGAAGGAAGGCCACAGCTGAGCAAATACTACATAGCTGGAGCTG
  |           || | |  |  |||||||||||||||||||||||||||||||||
CTGATCCAGGTGAGAGTACGGGGTACACAGCTGAGCAAATACTACATAGCTGGAGCTG
```

WT (>4bp overlap) 1 of 77 junctions

WT - 7bp overlap

```
ACACTGGACTGTTCTGAGCTGAGATGAGCTGGGGTGAGCTCAGCTATGCTACGCTGT
|||||||||||||||||||||||||||||||| |  ||        ||     |
ACACTGGACTGTTCTGAGCTGAGATGAGCTGGAGCTAGTATGAAGGTGGAGGTCCAG
 || |  |        |    |   ||||||||||||||||||||||||||||||||
GCAGTCAAGCTCAGCTACTACATGAGAGCTGGAGCTAGTATGAAGGTGGAGGTCCAG
```
